# Peripartum Management of Refractory Graves’ Thyrotoxicosis

**DOI:** 10.7759/cureus.102213

**Published:** 2026-01-24

**Authors:** Sonam Tshering, Ashutosh Kapoor, Sophie Buckley, Fareeha Rizvi

**Affiliations:** 1 Endocrinology and Diabetes, Barts Health NHS Trust, London, GBR; 2 Endocrinology and Diabetes, NMC Specialty Hospital, Dubai Investments Park, Dubai, ARE

**Keywords:** antithyroid drug resistance, beta blockade, peripartum, postpartum period, propylthiouracil, radioiodine therapy, refractory grave's disease, thyrotoxicosis

## Abstract

Graves’ disease (GD) is the most common cause of hyperthyroidism, characterized by autoimmune stimulation of the thyroid gland and typically managed with antithyroid drugs (ATDs), radioiodine (RAI) therapy, or surgery. While most patients respond to standard antithyroid therapy, a minority exhibit refractory thyrotoxicosis, posing significant clinical challenges. Pregnancy further complicates management due to immunological shifts, increased risk of adverse maternal and fetal outcomes, and limitations in therapeutic options. We present the case of a 39-year-old woman with severe, persistent Graves’ thyrotoxicosis refractory to high-dose propylthiouracil (PTU), whose clinical course was complicated by pregnancy and postpartum deterioration. Despite management with adequate doses of PTU, she remained biochemically hyperthyroid throughout pregnancy, developed gestational hypertension, and delivered a baby with a cleft palate. Postpartum, she suffered from depression and further deterioration of her thyrotoxicosis, with laboratory evidence of markedly elevated thyroid hormones and thyroid-stimulating hormone (TSH) receptor antibodies, despite directly observed high-dose PTU therapy. Definitive management options were limited by a highly vascular thyroid gland, significant myopathy, intolerance to cholestyramine, and psychiatric comorbidities. Surgery was deemed high risk, and RAI therapy was ultimately chosen, with careful pre- and post-treatment using high-dose propranolol to mitigate the risk of thyroid storm. The patient tolerated RAI without major complications and achieved euthyroidism at six months post-treatment, but relapsed at nine months, which may have been influenced by factors such as the long delay between diagnosis and RAI therapy, the presence of a large goiter, and the inability to achieve normal thyroid levels before treatment. This case emphasizes the complexities of managing refractory Graves’ thyrotoxicosis in the peripartum period, particularly when conventional therapies fail, and definitive interventions carry elevated risk. It also highlights the importance of a multidisciplinary, patient-centered approach and the need for individualized management strategies in challenging clinical scenarios.

## Introduction

Graves’ disease (GD) is an autoimmune disorder in which thyrotropin receptor antibodies (TRAb) stimulate the thyroid-stimulating hormone (TSH) receptor, increasing thyroid hormone production and release [1]. GD represents the leading cause of hyperthyroidism, occurring at a rate of 20 to 50 new cases per 100,000 individuals each year [2]. The clinical manifestations of GD depend on the patient’s age at onset, the severity, and the duration of hyperthyroidism [2]. Common symptoms, reported in over half of affected individuals, include unintentional weight loss, fatigue, heat intolerance, tremor, and palpitations [2]. Diffuse thyroid enlargement is the predominant finding; however, patients residing in regions with iodine deficiency frequently exhibit concomitant nodular goiter [2]. Graves’ ophthalmopathy occurs at an annual rate of 16 cases per 100,000 women and three cases per 100,000 men [2]. Thyroid dermopathy is identified in approximately 1-4% of patients with GD and is primarily associated with severe ophthalmopathy [2]. Diagnosis relies on clinical features and biochemical evidence of thyroid hormone excess [1]. If uncertainty persists after initial assessment, further investigations, such as TSH receptor antibody measurement, radioactive iodine uptake studies, or thyroidal blood flow assessment by ultrasonography, may be utilized, depending on local resources [1]. Management of Graves’ hyperthyroidism involves either suppressing thyroid hormone synthesis with antithyroid drugs (ATDs) or reducing thyroid tissue through radioactive iodine therapy or total thyroidectomy [3]. Although most patients respond to ATDs, resistance is described in rare cases [4,5]. Untreated or partially treated thyrotoxicosis is associated with weight loss, osteoporosis, atrial fibrillation, embolic events, muscle weakness, tremor, neuropsychiatric symptoms, and, rarely, cardiovascular collapse and death [1].

Pregnancy presents unique management challenges. Inadequate management of thyrotoxicosis increases the risk of adverse pregnancy outcomes, including miscarriage, gestational hypertension, preterm delivery, low birth weight, intrauterine growth restriction, stillbirth, thyroid storm, and maternal heart failure [6]. Therefore, women with thyrotoxicosis should achieve a stable euthyroid status prior to conception [6]. 

Psychiatric comorbidity is also more common in GD and can complicate treatment decisions and adherence [7]. Studies have shown that depression and anxiety scores were significantly elevated in individuals with GD compared with healthy controls, even during periods of active hyperthyroidism [7]. Collectively, these observations underscore the need for targeted clinical attention and tailored psychosocial support for patient subgroups at increased risk of enduring mood impairment [7].

This case details a woman whose GD remained inadequately controlled during pregnancy, leading to gestational hypertension and necessitating an early cesarean delivery. The newborn was affected by a cleft palate. Following delivery, the patient developed postpartum depression and experienced further deterioration of severe thyrotoxicosis, which proved resistant to propylthiouracil (PTU) therapy. Ultimately, she was successfully treated with radioactive iodine while still hyperthyroid, under close hospital supervision. The case highlights the need for a multidisciplinary, individualized approach in complex endocrine scenarios.

## Case presentation

Initial history

A 39-year-old female, diagnosed with GD in 2018, was started on carbimazole and transitioned to PTU during her first viable pregnancy in 2019, achieving satisfactory biochemical control. Her obstetric history included two miscarriages, the first occurring with normal thyroid function in 2017 and the second at the time of thyrotoxicosis diagnosis, as well as a prior ectopic pregnancy necessitating left salpingectomy.

Persistent hyperthyroidism (2020-2023)

**Figure 1:**
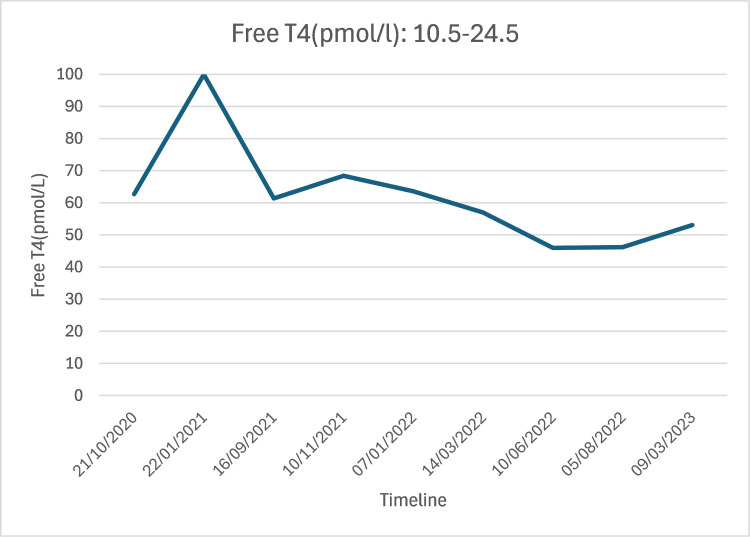
Persistent hyperthyroidism (2020-2023) Thyroid-stimulating hormone (TSH) was suppressed to <0.01 mU/L throughout. T4: thyroxine

Since 2020, the patient has experienced ongoing hyperthyroidism (Figure 1), characterized by intermittent non-attendance at clinic appointments and self-reported suboptimal adherence to carbimazole 40 mg once daily. In 2022, she was diagnosed with depression and started on sertraline 100 mg daily.

Second viable pregnancy and thyroid instability (2023)

While awaiting definitive thyroidectomy, the patient conceived unexpectedly in May 2023. At six weeks of gestation, she was found to be severely thyrotoxic (TSH <0.01 mU/L, free thyroxine (T4) 83.1 pmol/L), and her treatment was changed from carbimazole 40 mg OD to PTU 200 mg twice daily (BD) with dose adjustments throughout pregnancy. Despite reported adherence, she remained biochemically hyperthyroid throughout pregnancy (Table 1). She developed gestational hypertension and underwent cesarean delivery at 37 weeks. The neonate was born with a cleft palate and was not breastfed. Postpartum, her therapy was switched back to carbimazole 40 mg OD.

**Table 1:** Thyroid function tests during pregnancy T4: thyroxine; TSH: thyroid-stimulating hormone; T3: triiodothyronine; N/A: not available

Parameter	May 2023	August 2023	Oct 2023	Nov 2023	Dec 2023	Reference range
Free T4 (pmol/L)	83.1	48.2	33.9	28.9	30.1	10.5-24.5
TSH (mU/l)	<0.01	<0.01	<0.01	<0.01	<0.01	0.27-4.2
Free T3 (pmol/L)		24.1	N/A	N/A	11.4	3.1-6.8
TSH receptor antibodies (IU/L)	5	N/A	N/A	N/A	N/A	0-0.4

Postpartum deterioration

After delivery, she developed significant depressive symptoms requiring psychiatric admission. Six months postpartum, she reported worsening thyrotoxic symptoms, including palpitations, tremor, weight loss, insomnia, eye discomfort, difficulty climbing stairs, and difficulty holding the baby due to muscle weakness. Examination showed tachycardia (HR 110 bpm), mild proptosis (CAS = 1), goiter with bruit, and marked proximal myopathy.

Due to intolerance to high-dose carbimazole (nausea and vomiting), PTU was restarted at a high dose (200 mg TDS) alongside propranolol 40 mg TDS. Despite this, her laboratory investigations revealed persistently elevated free T4 (>100 pmol/L) and suppressed TSH (<0.01 mU/L) (Table 2). Based on this, after a multidisciplinary team discussion (MDT), she was admitted to the endocrine ward for direct observed therapy.

**Table 2:** Postpartum thyroid function tests T4: thyroxine; TSH: thyroid-stimulating hormone; T3: triiodothyronine; N/A: not available

Parameter	Feb 2024	May 2024	June 2024	July 2024	Reference range
Free T4 (pmol/L)	51.9	>100	>100	>100	10.5-24.5
TSH (mU/l)	<0.01	<0.01	<0.01	<0.01	0.27-4.2
Free T3 (pmol/L)	17.6	N/A	N/A	N/A	3.1-6.8

**Figure 2:**
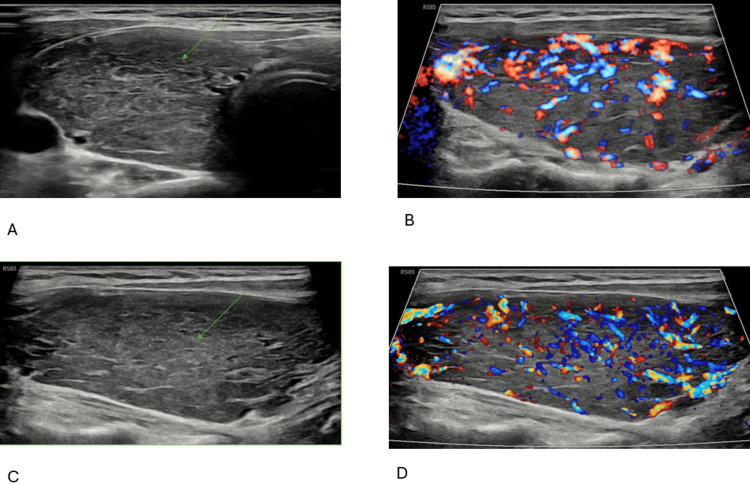
Thyroid ultrasound showed an enlarged, heterogeneous gland with florid vascularity A: plain ultrasound showing diffuse enlargement of the right thyroid lobe (arrow); B: color Doppler ultrasound showing increased vascularity of the right thyroid lobe; C: plain ultrasound showing diffuse enlargement of the left thyroid lobe (arrow); D: color Doppler ultrasound showing increased vascularity of the left thyroid lobe.

The echocardiogram showed normal LV function, mild left atrial dilation, mild to moderate mitral and mild tricuspid regurgitation, and intermediate probability of pulmonary hypertension. Thyroid ultrasound revealed an enlarged, heterogeneous gland with florid vascularity (Figure 2).

Despite observed therapy with PTU 200 mg TDS, she remained severely thyrotoxic with unmeasurable free T4 and free triiodothyronine (T3) levels above our assay (Table 3). It is also remarkable to note that her TSH receptor antibodies were unmeasurable above our assay (Table 3). The patient’s hemoglobin was normal at 137 g/L (reference: 120-150 g/L), with serum iron at 23 µmol/L (9-30 µmol/L), vitamin B12 at 724 ng/L (197-1000 ng/L), and folate at 6.5 mcg/L (3.9-9.9 mcg/L), all within reference ranges. Ferritin was slightly elevated at 504 mcg/L (13-150 mcg/L). Tissue transglutaminase IgA antibody was within range at 1 kU/L (0-5 kU/L), effectively ruling out celiac disease. Overall, there was no biochemical or clinical evidence of malabsorption in this case. Cholestyramine was trialed but discontinued due to gastrointestinal side effects. Steroids were considered but not used due to her psychiatric history.

**Table 3:** Thyroid function tests on observed PTU therapy in the hospital T4: thyroxine; TSH: thyroid-stimulating hormone; T3: triiodothyronine; N/A: not available; PTU: propylthiouracil

Parameter	22/06/24	25/06/25	30/06/25	02/07/24	16/07/25	Reference range
Free T4 (pmol/L)	>100	>100	>100	>100	>100	10.5–24.5
TSH (mu/l)	<0.01	<0.01	<0.01	<0.01	<0.01	0.27–4.2
Free T3 (pmol/L)	N/A	N/A	N/A	>50	N/A	3.1–6.8
TSH receptor antibodies	>30IU/L	N/A	N/A	N/A	N/A	0-0.4

Therapeutic decision‑making and radioiodine treatment

Given refractory thyrotoxicosis, two definitive options were discussed: surgery and radioiodine (RAI) therapy. Surgery was deemed high risk due to the patient’s vascular thyroid gland and myopathy, while RAI also carried a significant risk of thyroid storm because euthyroid status could not be achieved beforehand, and logistical challenges were present. Ultimately, the patient opted for RAI, receiving 600 MBq RAI (July 2024) under hospital supervision. Propranolol was titrated up to 240 mg TDS pre-RAI, and PTU was held for 24 hours before and after RAI. Propranolol was increased post-RAI to a maximum dose of 480 mg TDS and weaned gradually. Thyroid storm was prevented, and she was discharged after seven days post-RAI.

Outcome and follow‑up

Following RAI therapy, PTU was stopped after one month. The patient reached a euthyroid state six months later. However, at nine months post-treatment, she experienced a relapse of Graves’ thyrotoxicosis, as indicated by elevated thyroid hormone levels (free T4: 31.1 pmol/L, free T3: 7 pmol/L, TSH: <0.01) and persistently high TSH receptor antibody titers (TRAb: 29.93 IU/L). This recurrence necessitated restarting carbimazole at 15 mg daily and planning for a second course of RAI therapy.

## Discussion

The management of GD is well established, with ATDs such as carbimazole, methimazole, and PTU forming the cornerstone of initial therapy [3]. Remission rates with ATDs are generally reported at 50-55% after 12-18 months of treatment, and definitive therapy with RAI or thyroidectomy is recommended for relapsed or refractory cases [3]. True resistance to ATDs is exceedingly rare, with most cases of apparent treatment failure attributed to non-adherence or malabsorption [4,5]. In a series of patients with apparent PTU resistance, only one demonstrated true pharmacological resistance, while the majority were found to have normal drug absorption and metabolism [8]. In this case, there was no evidence of malabsorption clinically or biochemically, as the celiac screen, hemoglobin, and hematinics were all within normal limits. In the case described, persistent thyrotoxicosis despite directly observed high-dose PTU therapy supports the possibility of drug resistance, a phenomenon described only in isolated case reports. A published case report describes a 50-year-old woman with refractory thyrotoxicosis who failed to respond to carbimazole at doses up to 90 mg daily and subsequently to PTU at doses reaching 1200 mg per day, ultimately necessitating two courses of RAI therapy [9]. The patient exhibited a diffuse goiter with a bruit, mirroring features seen in our case. Her thyroid peroxidase antibody levels were markedly elevated (>1000 IU/ml; normal range 0-12), but TSH receptor antibody measurements were not available for comparison [9].

Psychiatric comorbidity is increasingly recognized in patients with GD [7], with studies demonstrating higher rates of depression and anxiety compared to healthy controls, even during periods of biochemical control [10]. Psychiatric illness can negatively impact treatment adherence and overall outcomes [11], as seen in our patient, who experienced depression and challenges with medication adherence during 2020-2023, contributing to early treatment failure. The birth of a child with a cleft palate, combined with ongoing, uncontrolled thyrotoxicosis after delivery, further exacerbated her depressive symptoms in the postpartum period. This underscores the importance of early identification and management of psychiatric symptoms in patients with complex endocrine disease [7]. 

GD often exacerbates in early pregnancy and postpartum, necessitating increased antithyroid therapy or resulting in relapse [12]. The underlying mechanism involves immunological shifts; pregnancy induces a shift towards Th2-dominant immunity, which reduces autoimmune activity, whereas the postpartum period triggers a rebound to Th1-dominant immunity, thereby amplifying autoimmune responses and raising the risk of disease exacerbation or relapse [13,14]. Postpartum, levels of TRAb may rise as a consequence of this rebound from pregnancy-associated immunosuppression [15]. Recent cohort studies have shown that patients with elevated TRAb at diagnosis are more likely to remain TRAb-positive during ATD therapy and face a higher risk of relapse after treatment withdrawal [16]. Notably, a TRAb level above 12 IU/l at diagnosis predicts a 60% chance of relapse at two years and 84% at four years [17]; the risk exceeds 90% if TRAb is greater than 7.5 IU/l at 12 months or above 3.85 IU/l at the end of therapy [17]. The clinical course of our patient closely reflected these findings, with a pronounced postpartum rise in TRAb and worsening thyrotoxicosis, in line with published evidence.

The management of refractory Graves’ thyrotoxicosis in the peripartum period presents significant therapeutic challenges due to increased risk of adverse maternal and fetal outcomes and limitations in therapeutic options. In this case, the patient developed gestational hypertension, which required a cesarean section at 37 weeks. ATDs carry teratogenic risks when used in early pregnancy [6]. Methimazole exposure during early pregnancy has been statistically linked to several congenital anomalies, including aplasia cutis, choanal and esophageal atresia, and various abdominal wall defects such as omphalocele, as well as abnormalities affecting the eyes, urinary system, and the ventricular septum of the heart, and affects 2-4% of children who have been exposed to methimazole in early pregnancy, especially during gestational weeks six to 10 [18,19]. The guidelines recommend PTU during the first trimester of pregnancy, then switching to methimazole or carbimazole for the remainder of the pregnancy, due to the risk of liver failure with PTU [3,6]. In this case, the patient was already six weeks pregnant when she discovered her pregnancy and had been taking carbimazole 40 mg daily; unfortunately, her baby was born with a cleft palate.

Achieving euthyroidism prior to definitive therapy is recommended to minimize the risk of thyroid storm during surgery or RAI administration [1,3]. However, this was not feasible due to persistent thyrotoxicosis despite high-dose, directly observed PTU therapy. Adjunctive therapies such as cholestyramine were trialed but discontinued due to gastrointestinal intolerance, and corticosteroids were avoided because of the patient’s severe postpartum depression. Surgical intervention was deemed high risk due to the highly vascular nature of the thyroid gland and the presence of significant myopathy, which increased the risk of intraoperative complications and anesthetic morbidity. RAI therapy was ultimately selected as the definitive treatment by the patient, but this approach also carried substantial risk. The inability to achieve a euthyroid state prior to RAI increased the potential for precipitating thyroid storm. To mitigate this, the patient was admitted for intensive inpatient monitoring and aggressive β-blockade, with propranolol titrated to high doses (up to 240 mg TDS) before and after RAI administration (up to 480 mg TDS on the third day). Despite these precautions, the risk of adverse outcomes remained elevated due to the severity of thyrotoxicosis.

Despite administration of 600 MBq of RAI, consistent with American Thyroid Association recommendations (370-555 Mbq) [1], the patient experienced a relapse nine months post-treatment. Meta-analytical data indicate that higher failure rates are associated with male sex, starting I-131 therapy more than six months after diagnosis, and previous anti-thyroid drug use [20]. Other factors linked to unsuccessful outcomes include raised free T4 levels, a 24-hour radioactive iodine uptake of 60.26% or more, and a thyroid volume of at least 35.77 mL [20]. These results suggest that certain clinical and biochemical features can help predict which patients are at greater risk of not responding to I-131 therapy and that early surgical intervention should be considered for those with a higher probability of treatment failure [20]. In this particular case, the relapse may have resulted from several contributing factors: the long interval between diagnosis and RAI treatment (seven years), prior anti-thyroid drug therapy, failure to achieve euthyroidism before RAI administration, and the presence of a diffuse goiter.

This case underscores the importance of a multidisciplinary approach, involving endocrinology, psychiatry, endocrine surgery, and anesthesiology, to navigate the therapeutic challenges posed by refractory GD in the postpartum period. Individualized management strategies, early identification of high-risk features, and proactive collaboration across specialties are essential to optimize outcomes in similarly complex scenarios.

In summary, this case aligns with and extends the existing literature by illustrating the rare occurrence of possible true ATD resistance, the impact of postpartum immunological rebound, and the critical role of multidisciplinary care in managing refractory GD complicated by psychiatric and social factors.

## Conclusions

This case highlights that, when conventional therapies fail to achieve euthyroidism and surgical intervention poses similar risks, RAI therapy administered under close inpatient supervision with robust beta-blockade can be a viable and effective treatment for severe refractory thyrotoxicosis. The persistence of profound thyrotoxicosis despite high-dose, directly observed PTU therapy suggests the rare possibility of true ATD resistance, warranting further clinical investigation. The pronounced postpartum rise in TRAb levels highlights the impact of immunological rebound in exacerbating disease severity, while the patient’s relapse and persistently elevated TRAb levels reinforce the need for ongoing monitoring. Importantly, this case also underscores the necessity of achieving euthyroidism prior to conception to minimize adverse pregnancy outcomes.

Overall, this case highlights the critical importance of a multidisciplinary, patient-centered approach in managing complex endocrine disorders, particularly in the postpartum period, where medical, psychological, and social factors intersect. Prompt identification of high-risk factors, multidisciplinary teamwork, and tailored management strategies are crucial to achieving the best possible outcomes in complex cases such as this.
